# Comparative Effect of Massage Therapy versus Kangaroo Mother Care on Body Weight and Length of Hospital Stay in Low Birth Weight Preterm Infants

**DOI:** 10.1155/2014/434060

**Published:** 2014-05-25

**Authors:** Priya Singh Rangey, Megha Sheth

**Affiliations:** S.B.B. College of Physiotherapy, V.S. Hospital Campus, Ellisbridge, Ahmedabad, Gujarat 380006, India

## Abstract

*Background*. Massage therapy (MT) and kangaroo mother care (KMC) are both effective in increasing the weight and reducing length of hospital stay in low birth weight preterm infants but they have not been compared. *Aim*. Comparison of effectiveness of MT and KMC on body weight and length of hospital stay in low birth weight preterm (LBWPT) infants. *Method*. 30 LBWPT infants using convenience sampling from Neonatal Intensive Care Unit, V.S. hospital, were randomly divided into 2 equal groups. Group 1 received MT and Group 2 received KMC for 15 minutes, thrice daily for 5 days. Medically stable babies with gestational age < 37 weeks and birth weight < 2500 g were included. Those on ventilators and with congenital, orthopedic, or genetic abnormality were excluded. Outcome measures, body weight and length of hospital stay, were taken before intervention day 1 and after intervention day 5. Level of significance was 5%. *Result*. Data was analyzed using SPSS16. Both MT and KMC were found to be effective in improving body weight (*P* = 0.001, *P* = 0.001). Both were found to be equally effective for improving body weight (*P* = 0.328) and reducing length of hospital stay (*P* = 0.868). *Conclusion*. MT and KMC were found to be equally effective in improving body weight and reducing length of hospital stay. *Limitation*. Long term follow-up was not taken.

## 1. Introduction

Preterm birth is defined as childbirth occurring at less than 37 completed weeks or 259 days of gestation [1].

Low birth weight (LBW), defined as weight at birth of less than 2500 grams irrespective of gestational age, has an adverse effect on child survival and development and may even be an important risk factor for adult diseases [2].

Newborn deaths currently account for approximately 40% of all deaths of children under five years of age in developing countries—the three major causes being birth asphyxia, infections, and complications due to prematurity and LBW [3]. Birth weight is a significant determinant of newborn survival. LBW is an underlying factor in 60–80% of all neonatal deaths. LBW infants are approximately 20 times more likely to die, compared with heavier babies [4].

Children who are born prematurely have higher rates of cerebral palsy, sensory deficits, learning disabilities, and respiratory illnesses compared with children born at term. The morbidity associated with preterm birth often extends to later life, resulting in enormous physical, psychological, and economic costs [5].

Researchers have provided hospitalized preterm infants with various forms of supplemental stimulation in an effort to enrich the environment of the neonatal intensive care unit (NICU) or to accelerate development [6, 7]. Two of the most widely studied interventions have been massage therapy and kangaroo mother care. In developing countries, financial and human resources for neonatal care are limited and hospital wards for LBW infants are often overcrowded [8]. KMC and MT are cost effective approaches that can be used by one and all irrespective of their financial status.

Massage is referred to as “a methodological touch intended to stimulate the baby.” A number of studies have shown the positive effects of massage therapy in preterm infants. These positive effects include weight gain, improved sleep/wake states, decreased stress, early discharge from the NICU, improved skin integrity, increased development of the sympathetic nervous system, and enhanced parent-infant bonding [9].

In 1978, Rey and Martinez proposed and developed Kangaroo mother care (KMC) at Instituto Materno Infantil in Santa Fe de Bogotá, Colombia, as an alternative to the conventional contemporary method of care for LBW infants. The term KMC is derived from similarities to marsupial care-giving. The mothers are used as “incubators” and as the main source of food and stimulation for LBW infants while they mature enough to face extrauterine life in similar conditions as those born at term [10]. Kangaroo mother care is defined as “Early, prolonged and continuous skin-to-skin contact between the mother and low birth weight infant both in the hospital and after discharge with exclusive breastfeeding and proper follow-up” [10]. Kangaroo mother care regularizes heart rate and respirations, deepens sleep and alert inactivity, reduces crying, prevents infections, shortens the neonatal hospital stay, enhances weight gain, improves physical growth and breastfeeding rates, decreases pain from heel prick procedure, and lessens maternal depression [8, 11–14].

Massage therapy (MT) and kangaroo mother care (KMC) are both effective in increasing the weight in low birth weight preterm infants and reducing the hospital stay. But still, they have not been compared to know which is more effective.

## 2. Aims and Objectives

The aims and objectives of this study are to compare the effectiveness of massage therapy and kangaroo mother care on weight gain and length of hospital stay in low birth weight preterm infants.

## 3. Materials and Methods

A quasi-experimental study was conducted with a convenience sample of 30 subjects at the NICU of V. S. Hospital in 2013. Infants born at gestational age of <37 weeks, having low birth weight, and medically stable were included and those who were medically unstable, had any congenital, orthopedic, or genetic abnormality, or were ventilated were excluded. Informed consent was taken from the parents. The infants were randomly divided in 2 groups with 15 infants in each group. Group 1 received 15 minutes of MT thrice daily for 5 days. Group 2 received at least 15 minutes of KMC thrice daily and it was continued later on as well by the mother with the physiotherapist in the NICU for 5 days. Body weight was taken before intervention on day 1 and after intervention on day 5, whereas length of hospital stay was calculated from the day of birth to discharge. Level of significance was kept at 5%.

MT was given according to the Field massage therapy protocol. Infants were massaged for 15 minutes, 3 times each day, at least 1 hour after being fed. Each massage session consisted of 5 minutes of tactile stimulation, 5 minutes of kinesthetic stimulation, and another 5 minutes of tactile stimulation. During the tactile stimulation the infant was placed in a prone (face down) position and given moderate pressure stroking with the bottom of the fingers of both hands. During the kinesthetic stimulation, the infant was placed in a supine (on back) position and led through passive flexion/extension actions [6]. For massage therapy, coconut oil was used as it has been found to be better than mineral oil [15].

During KMC the infant, wearing only a nappy (diaper), was placed between the mother's uncovered breasts. The mother was seated on a standard rocking chair, tilted at an angle of approximately 60°.

## 4. Results

Data was analyzed using SPSS version 16. Wilcoxon test was applied to determine whether there was significant difference within the groups. Mann Whitney-*U* test was applied to determine whether there was any significant difference between both groups or not. Both MT and KMC, respectively, were found to be effective in improving body weight (*P* = 0.001, *P* = 0.001) as shown in Table 1. However, both were found to be equally effective for improving body weight (*P* = 0.328) and reducing hospital stay (*P* = 0.868) as shown in Table 2.

## 5. Discussion

These findings show that MT and KMC promote weight gain and reduce hospital stay. There was an increase in body weight in the MT group similar to the findings of Dieter et al. who in 2003 studied that massage therapy leads to weight gain [16]. Dieter et al. in 2003 examined the effects of 5 days of massage therapy on the weight gain and sleep/wake behavior of hospitalized stable preterm infants and concluded that even 5 days of massage therapy were effective in improving weight and reducing sleep instead of 10 days that were practiced earlier [16].

It has been noticed that the neonates who gained more weight in the previous studies neither ingested more calories, nor spent more time sleeping, which might have allowed them more time to digest. In response to these findings Diego et al. in 2008 explored a theory that moderate pressure massage stimulates vagal activity (the activation of the vagal nerve is an index of parasympathetic nervous system activation), which leads to an increase in the release of digestive hormones and an increase in gastric motility.

Massage has also been shown to help neonates decrease stress behaviors and activities. The pacifying effect that massage has on preterm infants could benefit their health and reduce their length of time in the NICU. It may also desensitize the neonates to the stressful environment of the NICU by prolonging the time of parasympathetic activity (the resting, steady state, or nonstressed state of the autonomic nervous system). This in turn relates to increased vagal activity, which, as discussed earlier, leads to weight gain [17].

There was also a reduction in the length of hospital stay. The same findings were observed by Mendes and Procianoy in 2008. They studied the effect of maternal massage therapy on hospital stay in very low birth weight infants who were already submitted to skin-to-skin care and concluded that maternal massage therapy in very low birth weight infants decreases the length of hospital stay and the incidence of late-onset neonatal sepsis [18]. This reduction might be attributed to the improvement in body weight of the infant, improved sleep-wake states, improved immunity, and reduced stress behaviors and activity after massage therapy.

In the KMC group there was an increase in body weight and reduction in hospital stay. Roberts et al. in 2000 compared KMC with conventional cuddling care and found that KMC led to an improvement in body weight but it was equal to the weight gain observed in the conventional cuddling group [19]. They also observed that KMC leads to a reduction in the length of hospital stay. Cattaneo et al. also concluded that KMC infants have a higher mean daily weight gain and are discharged earlier compared to infants receiving conventional methods of care [20].

The weight gain in the KMC group might be due to improved breastfeeding rates, improved vagal tone, improved sleep cycles, and improved metabolic rates. Similarly the reduction in the hospital stay may be attributed to an overall decline in the infection rates and illnesses. Also, improved mother-infant bonding leads to a better health condition.

## 6. Conclusion

MT and KMC are both equally effective in improving weight and reducing hospital stay. MT and KMC can be used interchangeably as both are equally effective. In settings where professionals are not available to apply MT, KMC can be used in place of massage. KMC is also more community friendly as it does not require any special set-up or training. It can be given at any time according to the mother's wish. Also, the procedure can be performed by any other family member in absence of the mother.

*Limitations.* There are several factors that can have an effect on the outcome measures used in this study. Here, such factors like feeding amount and urine and stool output for body weight, basal metabolic rate, measures, and so forth were not monitored. Also, there are several measures to monitor the vagal activity like electroencephalography, electrogastrography, and so forth. But these measures are beyond the scope of physiotherapy. But still, they should also be monitored.

## Figures and Tables

**Table 1:** Comparison of means of body weight in Groups A and B.

Parameter	Group	Pre	Post	*Z* value	*P* value	Significance
Body weight (kgs)	A	1.53 ± 0.26	1.57 ± 0.25	−3.412	0.001	Yes
	B	1.46 ± 0.23	1.51 ± 0.22	−3.353	0.001	Yes

**Table 2:** Comparison of difference of means of Groups A and B for body weight and length of hospital stay.

Parameter	Group A	Group B	*U* value	*P* value	Significance
Body weight (gms)	45.3 ± 22.08	41.0 ± 29.83	89	0.328	No
Length of hospital stay (days)	22.13 ± 4.31	21.87 ± 3.33	108.5	0.868	No
